# Hepatotoxizität durch Albendazol: sichere Alternativen bei Echinokokkosetherapie

**DOI:** 10.1007/s00108-024-01796-y

**Published:** 2024-09-23

**Authors:** Laura Muana Wilhelm, Joschka Bachmann, Markus Cornberg, Heiner Wedemeyer, Bernd Heinrich

**Affiliations:** Klinik für Gastroenterologie, Hepatologie, Infektiologie und Endokrinologie, Medizinische Hochschule Hannover (MHH), OE 6810, Carl-Neuberg-Str. 1, 30625 Hannover, Deutschland

**Keywords:** *Echinococcus multilocularis*, Alveoläre Echinokokkose, Albendazolassoziierte Hepatitis, Albendazolassoziierte Toxizität, Mebendazol, *Echinococcus multilocularis*, Alveolar echinococcosis, Albendazole-associated hepatitis, Albendazole-associated toxicity, Mebendazole

## Abstract

Die Infektion mit *Echinococcus multilocularis* führt zum Krankheitsbild der alveolären Echinokokkose. Diese ist gekennzeichnet durch Ausbildung von alveolären Lebertumoren, welche im Verlauf meist nekrotisch zerfallen. Es kommt zur Ausbildung von Pseudozysten. Vor allem in frühen Stadien wird die kurative Resektion mit anschließender Langzeitbehandlung mit Albendazol empfohlen. Eine nicht seltene Komplikation unter Albendazoltherapie ist die Entwicklung einer Hepatitis. Wir präsentieren den seltenen Fall einer albendazolassoziierten Hepatitis bei nicht operabler *Echinococcus-multilocularis*-Infektion mit günstigem Verlauf durch Umstellung der Therapie auf Mebendazol.

## Abstract (en)

Infection with *Echinococcus multilocularis* leads to the clinical manifestation of alveolar echinococcosis. This is characterized by the formation of alveolar liver tumours, which usually disintegrate necrotically in the course of the disease. Pseudocysts are formed. Especially in the early stages, curative resection followed by long-term treatment with albendazole is recommended. However, the majority of patients are not amenable to curative surgery. In these cases, albendazole therapy is the first-choice treatment. We present a rare case of albendazole-associated hepatitis in a patient with inoperable *Echinococcus multilocularis* infection, with a favourable outcome following a change in treatment to mebendazole.

## Anamnese

Ein 72-jähriger Patient wurde bei extern erhobener positiver Echinokokkenserologie in der hepatologischen Sprechstunde vorstellig. Bei dem Patienten bestand seit acht Jahren ein lokal fortgeschrittenes, nicht metastasiertes Prostatakarzinom. Im Rahmen der diesbezüglichen Verlaufskontrollen war erstmals vor sieben Jahren ein neuer Leberherd festgestellt worden, welcher vor einem Jahr CT-gesteuert biopsiert wurde. Histologisch ergab sich kein Hinweis auf ein malignes Geschehen mit Nachweis von reichlich Nekrosen. Ein Jahr später zeigte sich dann ein positiver Befund in der Echinokokkenserologie und es erfolgte die Vorstellung in unserer Spezialambulanz. Zum Zeitpunkt der Vorstellung bestanden keinerlei Beschwerden. Auslandsaufenthalte wurden vor sieben Jahren in Spanien und Island, vor fünf Jahren in China und den USA und vor vier Jahren in Mexiko angegeben. Der Patient war beruflich im Bereich Maschinenbau tätig.

## Befund

**Abb. 1:**
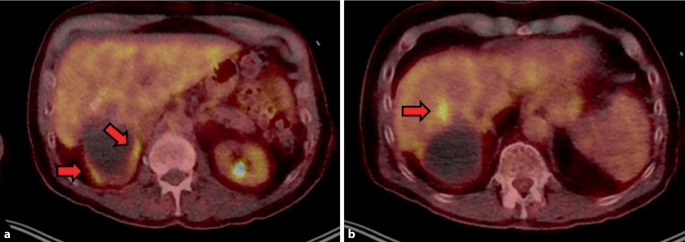
FDG-PET/CT bei Diagnosestellung: Entzündlich gesteigerter Glukosestoffwechsel im Randbereich der Zyste dorsal der Leber (**a**, *rote Pfeile*) und intrahepatisch in Segment VIII (**b**, *roter Pfeil*). CT-morphologisch intrahepatische Hypodensitäten an der V. cava, am ehesten vereinbar mit einer Pelottierung der V. cava durch eine Zyste

Im Labor zeigte sich ein leicht erhöhter CRP-Wert (8,7 mg/l) ohne Leukozytose. Das Gesamt-IgE war mit 3428 IU/ml erhöht. Es ergab sich keine Eosinophilie des Blutbilds. Das CA19‑9 war erhöht (47 kU/l [Norm: 27 kU/l]) bei ansonsten unauffälligen Tumormarkern. Ferner war die *Echinococcus*-Serologie mit einer *Echinococcus-multilocularis*-Infektion vereinbar (*Echinococcus*-IHA positiv [Titer 640], *Echinococcus-multilocularis*-Antigen ELISA positiv). In der Sonographie zeigte sich eine Hepatomegalie mit einem zystischen, teils verkalkten Lebertumor der Größe 14 × 10 × 8 cm, die Segmente VI, VII und VIII übergreifend. Weiterhin ergab sich der V. a. Infiltration der V. cava in Höhe des Leberhilus. Aufgrund einer implantierten Stimulationselektrode bei Morbus Parkinson war die Durchführung einer MRT-Untersuchung nicht möglich. Zur weiteren Diagnostik und zur Ermittlung der Krankheitsaktivität bei nun bereits eindeutigem V. a. Echinokokkose wurde daher eine FDG-PET/CT durchgeführt [1]. Es zeigte sich ein entzündlich gesteigerter Glukosestoffwechsel im Randbereich der bekannten Zyste dorsal der Leber sowie intrahepatisch in Segment VIII. Ferner präsentierten sich CT-morphologisch intrahepatische Hypodensitäten an der V. cava, am ehesten vereinbar mit einer Pelottierung der V. cava durch eine Zyste (Abb. 1).

## Diagnose

Gemäß der WHO-Definition ergab sich somit unter Berücksichtigung der klinischen Historie, der Bildgebung und der Serologie die „wahrscheinliche Diagnose“ („probable case“) einer *Echinococcus-multilocularis*-Infektion. Ein direkter Nachweis von *E. multilocularis* oder eine histologische Untersuchung und somit die Kriterien für eine bestätigte Diagnose („confirmed case“) lagen nicht vor [1]. Fern- und Lymphknotenmetastasen zeigten sich in keiner Bildgebung. Aufgrund des Verlaufs der Läsion entlang der großen hilusnahen Gefäße und des sonographischen Verdachts einer Infiltration der V. cava inferior am Leberhilus ist die Ausbreitung a.e. als P4 N0 M0 entsprechend und somit als Stadium IIIb gemäß WHO-Definition einzustufen („any liver lesion with extension along the vessels and the biliary tree; Vessels mean inferior vena cava, portal vein and arteries“; [2]).

## Therapie und Verlauf

**Abb. 2:**
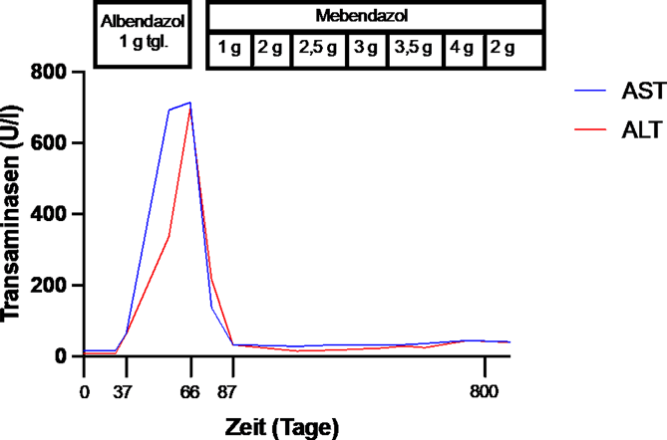
Transaminasenverlauf während des Behandlungszeitraums unter Albendazol- und Mebendazoltherapie

Entsprechend der Leitlinie waren die Diagnosekriterien für eine „wahrscheinliche Diagnose“ einer *Echinococcus-multilocularis*-Infektion erfüllt und somit eine Therapie indiziert. Es erfolgte der Beginn einer Anthelminthikatherapie mit dem aktuell präferierten Benzimidazol Albendazol mit einer Dosierung von 400 mg zweimal täglich (4 h-Zielspiegel: 1–3 µmol/l [1]). Parallel erfolgte zur Evaluierung eines kurativen Therapieansatzes eine chirurgische Vorstellung. Hier wurde eine Resektion nach mindestens 4‑wöchiger Einnahme von Albendazol und nach erneuter Bildgebung für möglich erachtet, jedoch aufgrund von Komorbiditäten sowie des Wunschs des Patienten nach konservativer Therapie zunächst zurückgestellt. 6 Wochen nach Beginn der Albendazoltherapie zeigten sich erstmals leicht steigende Transaminasen sowie eine steigende alkalische Phosphatase. Im kurzfristigen Verlauf zeigte sich ein progredienter Anstieg der Leberwerte mit führender GOT bis auf das 20fache der Norm (GOT 693 U/l, GPT 338 U/l, AP 164 U/l; Abb. 2). Klinisch bestanden Schwindel und Oberbauchschmerzen. Andere Ursachen einer Hepatitis, einschließlich viraler Hepatitis, toxischer Schädigungen durch andere Medikamente oder Substanzen sowie vaskulärer Ursachen wie Ischämie, wurden ausgeschlossen, sodass sich die Diagnose einer a.e. medikamentös-toxischen Hepatitis unter Albendazoltherapie stellen ließ. Einschränkungen der Lebersyntheseleistung und Entgiftungsfunktion zeigten sich zu keinem Zeitpunkt der Therapie. Auch das Blutbild wies keine Pathologien auf. Die Medikation wurde pausiert und innerhalb einer Woche kam es bereits zu einem deutlichen Abfall der Transaminasen. Innerhalb von 19 Tagen nach Pausierung der Medikation war eine vollständige laborchemische Remission zu verzeichnen (Abb. 2). Nach dieser akuten toxischen Hepatitis unter Albendazol wurde die Wiederaufnahme einer systemischen Therapie diskutiert. Mit dem Patienten wurden die bestehenden Therapieoptionen, einschließlich einer Reexposition mit Albendazol in einschleichender Dosierung, besprochen. Aufgrund der Schwere der Hepatotoxizität unter Albendazol und unter Berücksichtigung des Patientenwillens wurde schließlich eine Umstellung auf Mebendazol beschlossen. Diese wurde zunächst einschleichend mit initial 500 mg zweimal täglich dosiert (Zieldosis: 40–50 mg/kg/d in 3 Dosen, 4 h-Zielspiegel > 250 nmol/l [1, 3]). Nach einwöchiger Einnahme von 1 g Mebendazol täglich bestand weiterhin ein gutes Befinden mit unauffälligen Laborwerten, sodass die Dosis spiegeladaptiert schrittweise erhöht werden konnte (Abb. 2). Das IgE war kontinuierlich rückläufig (Erstvorstellung: 3428 IU/ml, ein Jahr nach Therapiebeginn: 2159 IU/ml, zwei Jahre nach Therapiebeginn: 1896 IU/ml).

**Abb. 3:**
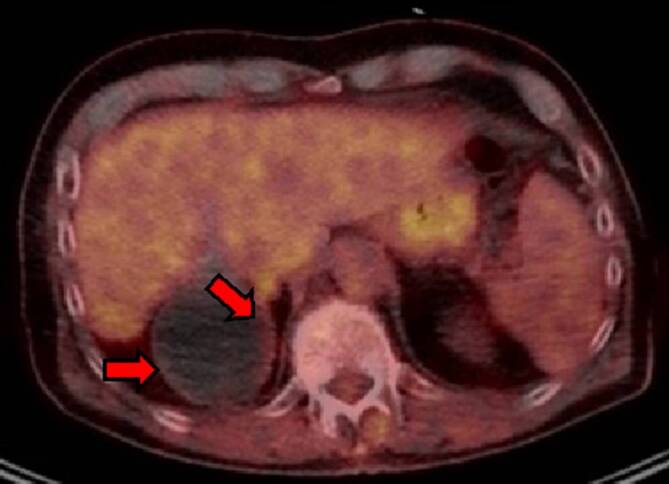
FDG-PET/CT 9 Monate nach Mebendazolbeginn unter 3,5 g Mebendazol täglich: In der größeren *Echinococcus*-Zyste dorsal der Leber deutlich regrediente, an der mediokaudalen Wand noch gering nachweisbare Aktivität (*rote Pfeile*)

9 Monate nach Therapiebeginn mit Mebendazol erfolgte eine FDG-PET/CT-Bildgebung unter 3,5 g Mebendazol täglich. Hier zeigte sich in der größeren Echinokokkuszyste dorsal der Leber eine deutlich regrediente, an der mediokaudalen Wand noch gering nachweisbare Aktivität (Abb. 3).

20 Monate nach Therapiebeginn zeigte die FDG-PET/CT unter 4 g Mebendazol täglich eine größenkonstante Echinokokkuszyste mit allenfalls flauer Stoffwechselsteigerung. Die Mebendazoldosis wurde bei rückläufigem IgE und tendenziell rückläufiger Aktivität in der Bildgebung insbesondere aufgrund des Patientenwillens bei hoher Tablettenanzahl (Mebendazol ist maximal in 500 mg-Dosierung verfügbar) und Notwendigkeit der häufigen Rezeptierung auf 2 g täglich halbiert. 3 Monate später zeigte sich auch hierunter das IgE weiterhin fallend, die Transaminasen normwertig und der sonographische Befund stabil. Ebenso erfolgte nach 24 Monaten eine Verlaufskontrolle des *Echinococcus*-IHA-Titers, welcher eine Reduktion von initial 640 auf einen Wert von 160 zeigte (Nachweisgrenze) und somit ebenso ein gutes Therapieansprechen dokumentierte.

## Diskussion

Die alveoläre Echinokokkose ist eine seltene Erkrankung (ca. 20–40 Fälle pro Jahr in Deutschland). *Echinococcus multilocularis* (sog. Fuchsbandwurm) befällt insbesondere die Leber, wobei es zu einem tumorartigen, infiltrativen Wachstum kommen kann. Der genaue Infektionsweg ist bisher nicht gänzlich geklärt. Der Großteil der Patienten mit alveolärer Echinokokkose ist bei Diagnosestellung nicht mehr kurativ operabel, sodass eine Langzeittherapie mit Benzimidazolen notwendig wird. Hierzu zählt sowohl Albendazol als auch Mebendazol [4]. Beide Präparate entfalten ihre Wirkung durch Schädigung zytosolischer Mikrotubuli mit konsekutiven Stoffwechselstörungen der Helminthen, wobei Mebendazol im Gastrointestinaltrakt schlechter resorbiert wird im Vergleich zu Albendazol [5]. Mögliche schwerwiegende Nebenwirkungen unter Benzimidazoltherapie umfassen in etwa 28,5 % der Fälle erhöhte Transaminasen. In einer Untersuchung zeigte sich in 6,9 % der Fälle eine schwere Hepatotoxizität, die einen Medikamentenwechsel oder eine Medikamentenpause erforderlich machte [6]. Auch Fälle von akutem Leberversagen sind dokumentiert. Eine milde Erhöhung der Transaminasen ist nach Pausierung der Medikation zwar reversibel, nach Reexposition mit z. B. Albendazol jedoch potenziell reproduzierbar [7]. Initial praktizierte Therapiepausen unter Albendazoltherapie waren aufgrund mangelnder Langzeittoxizitätsanalysen eingeführt worden, werden heute allerdings nicht mehr empfohlen [8]. Insbesondere bei Patienten mit inoperabler alveolärer Echinokokkose ist eine lebenslange antiinfektive Therapie kritisch und eine Therapieoption mit Mebendazol bei Albendazolunverträglichkeit sehr wertvoll [9]. Dieser Fall zeigt, dass Mebendazol für Patienten mit inoperabler alveolärer Echinokokkose nach albendazolassoziierter Hepatotoxizität eine wirksame und sichere Therapiealternative darstellen kann.

## Fazit für die Praxis

- Eine regelmäßige Überwachung der Leberwerte, des Blutbilds und auch der Medikamentenspiegel sollte bei Patienten unter Benzimidazoltherapie erfolgen.
- Bei Transaminasenanstieg während einer Therapie mit Albendazol sollte an eine albendazolassoziierte Toxizität gedacht werden.
- Das Auftreten einer Hepatitis unter Albendazoltherapie bedingt nicht zwangsläufig eine Toxizität unter Mebendazoltherapie.
- Ein Wechsel zu Mebendazol kann bei Patienten mit nichtoperabler alveolärer Echinokokkose und albendazolassoziierter Toxizität eine effektive alternative Therapieoption darstellen.
